# Supplementary material for: Large-scale photonic network with squeezed vacuum states for molecular vibronic spectroscopy
Source: Nat Commun. 2024 Jul 18;15:6057. doi: 10.1038/s41467-024-50060-2 (PMC11258230; doi:10.1038/s41467-024-50060-2)
Supplement: Supplementary file 2 — Supplementary information [file 41467_2024_50060_MOESM2_ESM.pdf]

Large-scale photonic network with squeezed vacuum states for molecular vibronic spectroscopy

H. H. Zhu, H. S. Chen, *et al.*

Supplementary Note 1: Theory on vibronic spectra with squeezed vacuum state

In this section, the scheme of vibronic spectra simulation with a squeezed vacuum state and a linear network is described. Figure 1 illustrates the simulation model, consisting of three main operations and measurements: squeezing operations (squeezer), rotation operations (Mach-Zehnder interferometers (MZI) network), and fock-based measurement with post-summation.

First, we denote the creation and annihilation operators of jth mode as $\hat{a}_j^\dagger$ and $\hat{a}_j$, and introduce the column vectors of m modes creation and annihilation operators as $\hat{\mathbf{a}}^\dagger = (\hat{a}_1^\dagger, \hat{a}_2^\dagger, ..., \hat{a}_m^\dagger)^T$, $\hat{\mathbf{a}} = (\hat{a}_1, \hat{a}_2, ..., \hat{a}_m)^T$ [1]. The Squeezer on mode j with a real parameter r_j is represented by a squeezing operator $\hat{S}_{r_j} = e^{\frac{1}{2}r_j^*\hat{a}_j^2 - \frac{1}{2}r_j\hat{a}_j^{\dagger 2}}$, whereby $\hat{S}_{r_j}\hat{a}_j\hat{S}_{r_j}^\dagger = \cosh(r_j)\hat{a}_j + \sinh(r_j)\hat{a}_j^\dagger$ [2]. We then denote $\hat{S}_{\mathbf{r}} = \otimes_{j=1}^m \hat{S}_{r_j}$ with $\mathbf{r} = (r_1, r_2, ...r_m)$. The MZI network encoded with matrix V corresponds to a rotation operator $\hat{R}_V$, whereby $\hat{R}_V^\dagger \hat{a}_j \hat{R}_V = \sum_{k=1}^m V_{jk}\hat{a}_k$.

In the simulation model, the output state of the optical network $|\phi\rangle = \hat{R}_V\hat{S}_{\mathbf{r}}|0\rangle$. With the photon number-resolving detection at the output of the network, the photon pattern on m modes $\mathbf{n} = (n_1, n_2, ..., n_m)$ is generated, where n_j is the number of photons in the jth mode. The probability of measuring a pattern $\mathbf{n}$ is equivalent to the overlap area of the output state $\hat{\rho}$ with the fock state projection $\hat{\mathbf{n}} = \bigotimes_{j=1}^m |n_j\rangle\langle n_j|$, which is expressed as

$$\begin{aligned} Pr(\mathbf{n}) &= \left|\langle \mathbf{n}| \hat{R}_V \hat{S}_{\mathbf{r}} |0\rangle\right|^2 \\ &= Tr(\hat{\rho}\hat{\mathbf{n}}). \end{aligned} \tag{1}$$

In the phase-space representation of quantum mechanics with Q and P functions, the probability is represented by the overlap integral of the Q function of output state $Q_{\hat{\rho}}(\boldsymbol{\beta})$ and P function of fock state projection $P_{\mathbf{n}}(\boldsymbol{\beta})$. As such, **Eq. (1)** is modified as

$$Pr(\mathbf{n}) = \pi^m \int d\boldsymbol{\beta} Q_{\hat{\rho}}(\boldsymbol{\beta}) P_{\mathbf{n}}(\boldsymbol{\beta}), \tag{2}$$

where $d\boldsymbol{\beta} = \prod_{j=1}^m d\beta_j d\beta_j^*$.

Subsequently, we calculate the Q function of the output state. The input-output relationship of the squeezer and the MZI network is $\hat{\mathbf{b}} = \hat{R}_V\hat{S}_{\boldsymbol{\lambda}}\hat{\mathbf{a}}\hat{S}_{\boldsymbol{\lambda}}^\dagger\hat{R}_V{}^\dagger$, where $\hat{\mathbf{a}}$ and $\hat{\mathbf{b}}$ are the annihilation operators of input and output [2]. With $\Lambda = diag(\boldsymbol{\lambda})$, the covariance matrix σ of output state is defined as $\sigma_{ij} = \frac{1}{2}\left\langle\left\{\xi_i, \xi_j^\dagger\right\}\right\rangle$, where ξ runs over all creation and annihilation operators of the output modes, which is given by

$$\sigma = \frac{1}{2}\begin{bmatrix} V & 0 \\ 0 & V^* \end{bmatrix}\begin{bmatrix} \cosh(\Lambda) & \sinh(\Lambda) \\ \sinh(\Lambda) & \cosh(\Lambda) \end{bmatrix}\begin{bmatrix} \cosh(\Lambda) & \sinh(\Lambda) \\ \sinh(\Lambda) & \cosh(\Lambda) \end{bmatrix}^\dagger\begin{bmatrix} V^\dagger & 0 \\ 0 & V^T \end{bmatrix}. \tag{3}$$

From the covariance matrix σ, we can construct the Q function of the output state by convolving the corresponding Wigner function

$$Q_{\hat{\rho}}(\boldsymbol{\beta}) = \frac{1}{\sqrt{|\pi\sigma_Q|}}\exp\left[-\frac{1}{2}\boldsymbol{\beta}_\nu^\dagger\sigma_Q^{-1}\boldsymbol{\beta}_\nu\right], \tag{4}$$

where $\sigma_Q = \sigma + I_{2m}/2$. I_{2m} is an identity matrix and $\boldsymbol{\beta}_\nu = [\beta_1, \beta_2, ...\beta_m, \beta_1^*, \beta_2^*, ...\beta_m^*]^T$.

The P function of the fock state projection is expressed as

$$P_{\mathbf{n}}(\boldsymbol{\beta}) = \prod_{j=1}^{m} \frac{e^{|\beta_j|^2}}{n_j!} \left(\frac{\partial^2}{\partial\beta_j \partial\beta_j^*} \right)^{n_j} \delta(\beta_j)\delta(\beta_j^*), \tag{5}$$

where $\delta(*)$ is an impulse function. By substituting **Eqs. (4)** and **(5)** into **Eq. (2)** and performing the integration,

$$Pr\left(\mathbf{n}\right) = \frac{1}{\mathbf{n}!\sqrt{|\sigma_Q|}} \prod_{j=1}^{m} \left(\frac{\partial^2}{\partial\beta_j \partial\beta_j^*} \right)^{n_j} \exp\left[\frac{1}{2} \boldsymbol{\beta}_\nu^\dagger \mathcal{A} \boldsymbol{\beta}_\nu \right] \Bigg|_{\boldsymbol{\beta}_\nu = 0}, \tag{6}$$

where $\mathcal{A} = \begin{bmatrix} 0 & I_m \\ I_m & 0 \end{bmatrix} [I_{2m} - \sigma_Q^{-1}] = \begin{bmatrix} A & 0 \\ 0 & A^* \end{bmatrix}$ with $A = V \tanh\left(\mathbf{r}\right) V^T$.

We then expand the derivatives in **Eq. (6)** using Faà di Bruno's formula [3], which gives an expansion equation for the n-th derivative composition $f(g(t))$ in terms of Bell polynomial $B_{n,k}(x)$, *i.e.*,

$$d^n f(g(t)) = \sum_{k=0}^{n} (d^k f)(g(t) B_{n,k}(dg(t), d^2 g(t), ...)), \tag{7}$$

in which the integral is exactly quadratic to the hafnian function [4].

$$\begin{aligned} Pr\left(\mathbf{n}\right) &= \frac{1}{\mathbf{n}!\sqrt{|\sigma_Q|}} \sum_{M \in PMP(\boldsymbol{n},\boldsymbol{n})} \prod_{(i,j) \in M} \mathcal{A}_{i,j}^{\mathbf{n}} \\ &= \frac{1}{\mathbf{n}!\sqrt{|\sigma_Q|}} |haf\left(A^{\mathbf{n}}\right)|^2, \end{aligned} \tag{8}$$

where $haf(\cdot)$ is the hafnian function, PMP is the set of perfect matching permutations. $A^{\mathbf{n}}$ is a submatrix of A depending on the measured output pattern $\mathbf{n}$. The generation rules of the submatrix are as follows

(1) The obtained array $S^{\mathbf{n}}$ is defined as

$$S^{\mathbf{n}} = \underbrace{1, ..., 1}_{n_1 times}, \underbrace{2, ..., 2}_{n_2 times}, ..., \underbrace{m, ...m}_{n_m times}. \tag{9}$$

For instance, if $\mathbf{n} = (1, 3, 0, 2)$, then

$$S^{(1,3,0,2)} = [1, 2, 2, 2, 4, 4]. \tag{10}$$

(2) The matrix $A^{\mathbf{n}}$ is produced by using the array $S^{\mathbf{n}}$ as $A_{ij}^{\mathbf{n}} = A_{S_i^{\mathbf{n}} S_j^{\mathbf{n}}}$.

For example, if

$$A = \begin{bmatrix} A_{1,1} & A_{1,2} & A_{1,3} & A_{1,4} \\ A_{2,1} & A_{2,2} & A_{2,3} & A_{2,4} \\ A_{3,1} & A_{3,2} & A_{3,3} & A_{3,4} \\ A_{4,1} & A_{4,2} & A_{4,3} & A_{4,4} \end{bmatrix}, \tag{11}$$

then,

$$A^{(1,3,0,2)} = \begin{bmatrix} A_{1,1} & A_{1,2} & A_{1,2} & A_{1,2} & A_{1,4} & A_{1,4} \\ A_{2,1} & A_{2,2} & A_{2,2} & A_{2,2} & A_{2,4} & A_{2,4} \\ A_{2,1} & A_{2,2} & A_{2,2} & A_{2,2} & A_{2,2} & A_{2,2} \\ A_{2,1} & A_{2,2} & A_{2,2} & A_{2,2} & A_{2,2} & A_{2,2} \\ A_{4,1} & A_{4,2} & A_{4,2} & A_{4,2} & A_{4,4} & A_{4,4} \\ A_{4,1} & A_{4,2} & A_{4,2} & A_{4,2} & A_{4,4} & A_{4,4} \end{bmatrix}. \tag{12}$$

The first row and column of A are repeated only once in $A^{(1,3,0,2)}$, the second one is repeated for three times ($n_2 = 3$), the third one does not appear since $n_3 = 0$, and the last one is repeated twice ($n_4 = 2$). It is also noted that $A^{\mathbf{n}}$ is symmetric if A is symmetric.

Up to here, the relationship between the hafnian function and the probability of measuring an output pattern from a squeezed vacuum state and a linear network is obtained. In the next step, we will further develop the connection between the molecular vibronic transitions and the hafnian. Within the Born-Oppenheimer approximation and the Condon approximation [5–8], the intensity of vibronic transition is defined as the square of the overlap integral between the vibrational states of different electronic states (**Fig. 1b**), which is known as the Franck-Condon factors (FCF). The coordinate space representations of the vibrational states are then obtained by projecting on the position operator eigenstates $|Q\rangle$ and $|Q'\rangle$, where Q and Q' are the mass-weighted normal coordinate space of the final and initial states, respectively, which are defined by

$$R - R_0 = M^{-1/2} L Q \tag{13a}$$

$$R' - R'_0 = M^{-1/2} L' Q', \tag{13b}$$

where R and R' denote the Cartesian displacement coordinates with respect to the reference configurations coinciding with the equilibrium structures R_0 and R'_0, respectively, both of which have an Eckart axis system attached. L and L' denote the normal modes of the initial and final states analyzed in the normal coordinates, and M is the diagonal matrix of atomic masses.

When the transformation between the Eckart axis systems of the final and initial states is an identity, the relation between the initial and final dimensionless normal coordinate is expressed as [5]

$$\begin{aligned} \hat{Q}' &= L'^t L \hat{Q} + L'^t M^{1/2} \left(R_0 - R'_0 \right) \\ &= U \hat{Q} + \mathbf{d}, \end{aligned} \tag{14}$$

which is the Duschinsky relation [9], where U is the Duschinsky matrix describing rotations in normal coordinate space and $\mathbf{d}$ is the shift of the origin along the normal coordinates. Under harmonic approximation, two sets of quantum harmonic oscillators (QHO) are correlated by the Duschinsky relation, which can be expressed as a

multidimensional Bogoliubov transformation as follows [10]:

$$\hat{\mathbf{a}}'^{\dagger} = \frac{1}{2}\left(J - (J^T)^{-1}\right)\hat{\mathbf{a}} + \frac{1}{2}\left(J + (J^T)^{-1}\right)\hat{\mathbf{a}}^{\dagger} + \frac{1}{\sqrt{2}}\mathbf{K}, \tag{15}$$

where J and $\mathbf{K}$ are defined as

$$\begin{aligned} J &= \Omega' U \Omega^{-1}, \\ \mathbf{K} &= \hbar^{-1/2}\Omega'\mathbf{d}, \\ \Omega' &= \text{diag}\left(\sqrt{\omega_1'}, ..., \sqrt{\omega_m'}\right), and \\ \Omega \ &= \text{diag}\left(\sqrt{\omega_1}, ..., \sqrt{\omega_m}\right), \end{aligned} \tag{16}$$

The notation 'diag' denotes a diagonal matrix, $\{\omega_k'\}$ and $\{\omega_l\}$ are the harmonic angular frequencies of the final and initial states. Doktorov *et al.* [11] defined a unitary operator $\hat{U}_{Dok}$, which performs the Duschinsky rotation as $\hat{\mathbf{a}}'^{\dagger} = \hat{U}_{Dok}^{\dagger}\hat{\mathbf{a}}^{\dagger}\hat{U}_{Dok}$. The Doktorov operator can be further decomposed as $\hat{U}_{Dok} = \hat{D}_{\boldsymbol{\alpha}}\hat{R}_{C_L}\hat{S}_{\boldsymbol{\lambda}}\hat{R}_{C_R'}$, where $C_L\text{diag}(\boldsymbol{\lambda})C_R' = J$ is the singular value decomposition (SVD) of J and $\boldsymbol{\alpha} = \frac{1}{\sqrt{2}}\mathbf{K}$ [10].

Note that the Doktorov transformation can be used to map any operator between the initial and final energy surface[6]. Thus any initial vibronic state $|\psi_{in}\rangle$ is transformed into $|\psi_{out}\rangle = \hat{U}_{Dok}|\psi_{in}\rangle$. Explicitly, the transition probability, which is called FCF, at 0 K is obtained with the initial vacuum state $|\mathbf{0}\rangle$, *i.e.*,

$$\text{FCF}(\mathbf{n}) = \left|\langle\mathbf{n}|\,\hat{U}_{Dok}\,|\mathbf{0}\rangle\right|^2. \tag{17}$$

With an inner product ($\omega_{vib} = \sum_k \omega_k' n_k$), each FCF gives an attached transition frequency. The Franck-Condon profile (FCP) at 0 K is obtained as

$$\text{FCP}(\omega_{vib}) = \sum_{n}^{\infty}\left|\langle\mathbf{n}|\,\hat{U}_{Dok}\,|\mathbf{0}\rangle\right|^2 \delta(\omega_{vib} - \sum_k \omega_k' n_k). \tag{18}$$

Then, the FCF is calculated with the Q and P functions as

$$\begin{aligned} \text{FCF}(\mathbf{n}) &= \left|\langle\mathbf{n}|\,\hat{D}_{\boldsymbol{\alpha}}\hat{R}_{C_L}\hat{S}_{\boldsymbol{\lambda}}\hat{R}_{C_{R'}}\,|\mathbf{0}\rangle\right|^2 \\ &= \pi^m \int d\boldsymbol{\beta} Q_{\hat{\rho}'}(\boldsymbol{\beta})P_{\mathbf{n}}(\boldsymbol{\beta}). \end{aligned} \tag{19}$$

Here, to distinguish with the displacement parameter $\boldsymbol{\alpha}$, we denote $\boldsymbol{\beta}$ to represent the integral element in the coherent state representation. Then, the covariance matrix σ' of the output state is $\sigma_{ij}' = \frac{1}{2}\left\langle\left\{\xi_i, \xi_j^{\dagger}\right\}\right\rangle - \alpha_i\alpha_j^*$, where ξ runs over all creation and annihilation operators of the output modes. The Q function with displacement is given by

$$\begin{aligned} Q_{\hat{\rho}'}(\boldsymbol{\beta}) &= \frac{1}{\sqrt{|\sigma_Q|}}\exp\left[-\frac{1}{2}(\boldsymbol{\beta}_\nu - \boldsymbol{\alpha}_\nu)^{\dagger}\boldsymbol{\sigma}_Q^{-1}(\boldsymbol{\beta}_\nu - \boldsymbol{\alpha}_\nu)\right] \\ &= \frac{1}{\sqrt{|\sigma_Q|}}\exp\left[-\frac{1}{2}\boldsymbol{\alpha}_\nu^{\dagger}\boldsymbol{\sigma}_Q^{-1}\boldsymbol{\alpha}_\nu - \frac{1}{2}\boldsymbol{\beta}_\nu^{\dagger}\sigma_Q^{-1}\boldsymbol{\beta}_\nu + \boldsymbol{\zeta}_\nu^T\boldsymbol{\beta}_\nu\right], \end{aligned} \tag{20}$$

where $\sigma_Q = \sigma' + I_{2m}/2$, $\boldsymbol{\zeta}_\nu^T = \boldsymbol{\alpha}_\nu^\dagger \sigma_Q^{-1}$, $\boldsymbol{\alpha}_\nu = [\alpha_1, \alpha_1, ...\alpha_m, \alpha_1^*, \alpha_2^*, ...\alpha_m^*]^T$, and $\boldsymbol{\beta}_\nu = [\beta_1, \beta_2, ...\beta_m, \beta_1^*, \beta_2^*, ...\beta_m^*]^T$.

We define $\mathcal{B} = \begin{bmatrix} 0 & I_m \\ I_m & 0 \end{bmatrix} [I_{2m} - \sigma_Q^{-1}] = \begin{bmatrix} B & 0 \\ 0 & B^* \end{bmatrix}$ with $B = C_L \tanh(\Lambda) C_L{}^T$, where C_L is the rotation matrix. Then, we obtain $\boldsymbol{\zeta} = \boldsymbol{\alpha} - B\boldsymbol{\alpha}^*$. The P function is the same as **Eq. (5)**. After substituting the Q function and the P function into **Eq. (19)** and performing the integration, it yields

$$\begin{aligned} \mathrm{FCF}\,(\mathbf{n}) &= \mathcal{N} \prod_{j=1}^{m} \left(\frac{\partial^2}{\partial \beta_j \partial \beta_j^*} \right)^{n_j} \exp\left[-\frac{1}{2} \boldsymbol{\beta}_\nu^T \mathcal{B} \boldsymbol{\beta}_\nu + \boldsymbol{\zeta}_\nu^T \boldsymbol{\beta}_\nu \right] \Bigg|_{\boldsymbol{\beta}_\nu = 0} \\ &= \mathcal{N} \sum_{\mathbf{l}_1=\mathbf{0}}^{\mathbf{n}} \sum_{\mathbf{l}_2=\mathbf{0}}^{\mathbf{n}} C_{\mathbf{n}}^{\mathbf{l}_1} C_{\mathbf{n}}^{\mathbf{l}_2} \left(\prod_k \zeta_k{}^{l_{1k}} \zeta_k{}^{*l_{2k}} \right) haf(\mathcal{B}^{\mathbf{n}-\mathbf{l}_1, \mathbf{n}-\mathbf{l}_2}) \\ &= \mathcal{N} \left| \sum_{\mathbf{l}=\mathbf{0}}^{\mathbf{n}} \left[C_{\mathbf{n}}^{\mathbf{l}} \left(\prod_k \zeta_k{}^{l_k} \right) haf(B^{\mathbf{n}\text{-}\mathbf{l}}) \right] \right|^2, \end{aligned} \tag{21}$$

where $\mathcal{N}$ is a normalized constant and $C_{\mathbf{n}}^{\mathbf{l}}$ denotes the binomial coefficient. Given that $\mathcal{B}$ is formed by duplicating matrix B, the FCF in the second line of **Eq. (21)** can be expressed as the relation to the hafnian of matrix B, as demonstrated in the final line of **Eq. (21)**.

According to **Eq. (21)**, the FCF is represented as a weighted summation of hafnian functions. However, it is not sufficient to establish a bosonic sampler for effectively obtaining the molecular vibronic spectra under this formula. This is because the hafnian value of each submatrix $B^{\mathbf{l}}$ contribute to the FCF ($\mathbf{n}$) for each $\mathbf{n} \geq \mathbf{l}$. This implies that even if one has access to the group output of the hafnian values of all submatrices generated by matrix B (which can be efficiently obtained using a bosonic sampler), reconstructing the corresponding molecular vibronic spectra is still difficult due to the need for computing an exponentially large number of spectral lines. Thus, we propose to represent the displacement parameters in terms of extended matrix elements to ensure that each hafnian value of a submatrix only contributes to a single FCF, and theoretically build the connection between FCF in **Eq. (21)** and the hafnian function of the extended matrix A as

$$\mathrm{FCF}\,(\mathbf{n}) = \mathcal{N} \left| \sum_{\mathbf{l}=\mathbf{0}}^{\mathbf{n}} \frac{1}{\mathbf{l}!} haf\left(A^{(\mathbf{n},\mathbf{l})} \right) \right|^2, \tag{22}$$

where A is an expanded matrix that codes the displacement information into the generated elements, i.e.,

$$A = \begin{bmatrix} B & Z \\ Z & 0 \end{bmatrix}, \tag{23}$$

where

$$
\begin{aligned}
B &= C_L \tanh(\Lambda)\, C_L{}^T, \\
\boldsymbol{\zeta} &= \boldsymbol{\alpha} - B\boldsymbol{\alpha}^*, \\
Z &= diag(\boldsymbol{\zeta}), \\
\Lambda &= \mathrm{diag}(\boldsymbol{\lambda}), \\
(\mathbf{n}, \mathbf{l}) &= (n_1, n_2, ..., n_m, l_1, l_2, ..., l_m).
\end{aligned}
\tag{24}
$$

Here, C_L, $\boldsymbol{\lambda}$ and $\boldsymbol{\alpha}$ are corresponded to the Doktorov transformation as $\hat{U}_{Dok} = \hat{D}_\alpha \hat{R}_{C_L} \hat{S}_{\boldsymbol{\lambda}} \hat{R}_{C_{R'}}$, $(\mathbf{n}, \mathbf{l})$ is an output pattern, $A^{(\mathbf{n},\mathbf{l})}$ is a submatrix about $(\mathbf{n}, \mathbf{l})$ generated by A, and $\boldsymbol{\zeta}$ is the vector formed by $\boldsymbol{\zeta} = \boldsymbol{\alpha} - B\boldsymbol{\alpha}^*$. **Eq. (22)** will be substantiated in the following section.

The $haf\left(A^{(\mathbf{n},\mathbf{l})}\right)$ is expressed as

$$
\begin{aligned}
haf\left(A^{(\mathbf{n},\mathbf{l})}\right) &= \sum_{M \in PMP(\boldsymbol{n},\boldsymbol{l})} \prod_{(i,j) \in M} A_{i,j} \\
&= \left(\prod_k \frac{n_k!}{(n_k - l_k)!} A_{k,k+n}^{l_k}\right) \left[\sum_{M \in PMP(\boldsymbol{n}-\boldsymbol{l},\mathbf{0})} \prod_{(i,j) \in M} A_{i,j}\right] \\
&= \left(\prod_k \frac{n_k!}{(n_k - l_k)!} \zeta_k{}^{l_k}\right) \left[\sum_{M \in PMP(\textbf{n-l})} \prod_{(i,j) \in M} B_{i,j}\right] \\
&= \frac{\mathbf{n}!}{(\mathbf{n} - \mathbf{l})!} \left(\prod_k \zeta_k{}^{l_k}\right) \left[\sum_{M \in PMP(\textbf{n-l})} \prod_{(i,j) \in M} B_{i,j}\right] \\
&= \frac{\mathbf{n}!}{(\mathbf{n} - \mathbf{l})!} \left(\prod_k \zeta_k{}^{l_k}\right) haf(B^{\textbf{n-l}}).
\end{aligned}
\tag{25}
$$

Then,

$$
\begin{aligned}
\sum_{\mathbf{l}=\mathbf{0}}^{\mathbf{n}} \frac{1}{\mathbf{l}!} haf\left(A^{(\mathbf{n},\mathbf{l})}\right) &= \sum_{\mathbf{l}=\mathbf{0}}^{\mathbf{n}} \left[\frac{\mathbf{n}!}{(\mathbf{n} - \mathbf{l})!\mathbf{l}!} \left(\prod_k \zeta_k{}^{l_k}\right) haf(B^{\textbf{n-l}})\right] \\
&= \sum_{\mathbf{l}=\mathbf{0}}^{\mathbf{n}} \left[C_{\mathbf{n}}^{\mathbf{l}} \left(\prod_k \zeta_k{}^{l_k}\right) haf(B^{\textbf{n-l}})\right].
\end{aligned}
\tag{26}
$$

Therefore, by substituting **Eq. (26)** into **Eq. (21)**, the relation between the FCF and hafnian of the extended matrix A via **Eq. (22)** is obtained. Then, the vibronic spectral profile called the FCP, is obtained by computing the corresponding FCF, *i.e.*,

$$
\mathrm{FCP}(\omega_{vib}) = \sum_{n}^{\infty} \mathrm{FCF}\,(\mathbf{n})\, \delta(\omega_{vib} - \sum_k \omega'_k n_k). \tag{27}
$$

The FCF integral is decomposed into a weighted summation of hafnian functions based on the involvement of the displacement operation for each mode in **Eq. (22)**. It is observed that, under two specific conditions below, it is possible to further refine the FCP approximation by ignoring the positive-definiteness of the hafnian function in

Eq. (22) and approximating the FCF using absolute values. These two conditions are: (1) the dominant terms have the same signs in the summation for wavenumbers with large FCF values, exceeding a normalized threshold of 0.2 for the molecules under discussion; (2) conversely, the absolute value of each term in the summation diminishes for wavenumbers with low FCF values, falling below a normalized threshold of 0.05 for the molecules discussed. Fortunately, these conditions are nearly met in most molecular systems, facilitating the implementation of the sign approximation. It can be explained that the resulting FCF value is large when terms in the summation closely align with the molecule's parameters, indicating dominance. As a comparison, for wavenumbers with small FCF values, no single term fully matches the molecule's parameters, resulting in each term's absolute value being small and contributing to the overall diminution of FCF values. To verify this assumption, the FCF is calculated numerically using a simplified equation, whereby only one term with the maximum value is retained in the summation, as shown in **Supplementary Figure 1**. The fidelity between the theoretical FCP and the reconstructed profile using the simplified equation is found to be high. This is achieved by randomly generating the transformation matrices U, displacement vector **K**, and vibrational frequencies ω and ω'. As shown in the red bar of **Supplementary Figure 1b**, it is evident that, when **K** is small, the dominant term is associated with small **l**, whereas, for larger values of **K**, the dominant term shifts to large **l**. This observation suggests that, among various combinations where each mode's displacement or squeezing operation exerts an influence, only one particular case has a dominant effect on the vibronic transition. It can be explained that, in the summation process, the variation in **l** adjusts the extent of involvement of the displacement vector **K**, and the value of **K** significantly influences each term in the summation [**Eq. (26)**]. Consequently, when **l** takes values at a pertinent position correlated with **K**, this term's magnitude becomes substantially greater than the others. This leads to the summation in **Eq. (22)** being typically dominated by one term. Therefore, we can disregard the positive-definiteness of the hafnian function and approximate the FCF using absolute values.

It should be noted that, however, there are also a few exceptional molecules with highly unique structures that defy the approximation in our method. For example, considering a virtual molecule with $B = \begin{pmatrix} 0 & 1 \\ 1 & 0 \end{pmatrix}$ and $Z = \begin{pmatrix} 1 & 0 \\ 0 & -1 \end{pmatrix}$, our approximation would yield a high error and low reconstructed fidelity due to its failure to meet the above conditions wherein the absolute value of two terms in the summation possess identical values but opposite signs, consequently leading to a small FCF value. Nonetheless, such molecules with matrices B and Z that adhere to this particular form are exceedingly rare, with none discovered among the 14,000 random molecules illustrated in **Supplementary Figure 2**. This rarity enables us to employ this approximation method for the molecular simulation with effectiveness and generality.

After disregarding the positive definition of the hafnian function, we further deduce that the approximated FCF

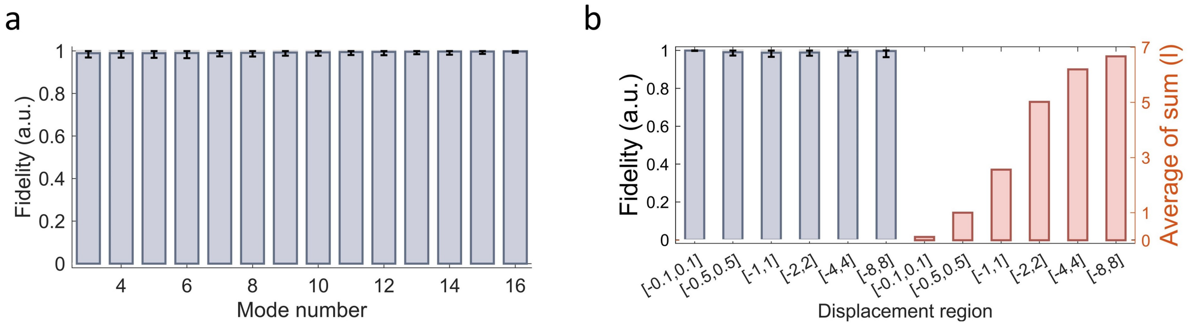

Supplementary Figure 1: **a** Statistical fidelities for different molecular mode sizes. **b** Statistical fidelities and average of selected $\mathbf{l}$ for different displacement regions. Blue bar is the statistical fidelity from the approximation by keeping the term with the maximum value in the summation in **Eq. (22)**. Red bar is the average of selected terms $\mathbf{l}$ that contribute to the maximum value of the summation in **Eq. (22)**.

at 0 K is expressed as

$$\begin{aligned}\widetilde{\mathrm{FCF}}\left(\mathbf{n}\right) &= \mathcal{N}\left|\sum_{\mathbf{l}=\mathbf{0}}^{\mathbf{n}}\frac{1}{\mathbf{l}!}\left|haf\left(A^{(\mathbf{n},\mathbf{l})}\right)\right|\right|^{2} \\ &= \mathcal{N}'\left|\sum_{\mathbf{l}=\mathbf{0}}^{\mathbf{n}}\left(\frac{Pr\left(\mathbf{n},\mathbf{l}\right)}{\mathbf{l}!}\right)^{\frac{1}{2}}\right|^{2},\end{aligned} \tag{28}$$

where $\mathcal{N}'$ is a normalization constant, and $Pr(\mathbf{n},\mathbf{l})$ is the probability to measure an output pattern $(\mathbf{n},\mathbf{l})$. In the experiment, after computing the Takagi-Autonne decomposition of A, we obtain the unitary $\tilde{U}_C$ and the squeezing value $\tilde{\mathbf{r}}$. By encoding these parameters into our boson sampling circuit with the photon number resolving measurement. The results of the approximate vibronic spectra are finally obtained as

$$\widetilde{\mathrm{FCP}}(\omega_{vib}) = \sum_{\mathbf{n}}^{\infty}\widetilde{\mathrm{FCF}}\left(\mathbf{n}\right)\delta(\omega_{vib} - \sum_{k}\omega_k' m_k). \tag{29}$$

The simulation about the fidelity of the profile using the approximated FCP in **Eq. (29)** is depicted in **Supplementary Figure 2**. We randomly generate a set of 14000 virtual molecules, and distribute them across 14 mode sizes. Each mode comprises 1000 molecules. The fidelity of vibronic spectra between the theory and our approximation distribution of randomly selected 100 arbitrary virtual molecular spectra per mode size (different colors belong to different dimensions) is displayed in **Supplementary Figure 2a**. It can be seen that the average fidelities (dashed line) do not decrease with the increasing mode size, and the measured average fidelities are always higher than 0.96. We further summarize the statistical accuracies for 14 different mode sizes in **Supplementary Figure 2b**, and verify that the approximation in **Eq. (29)** is reasonable.

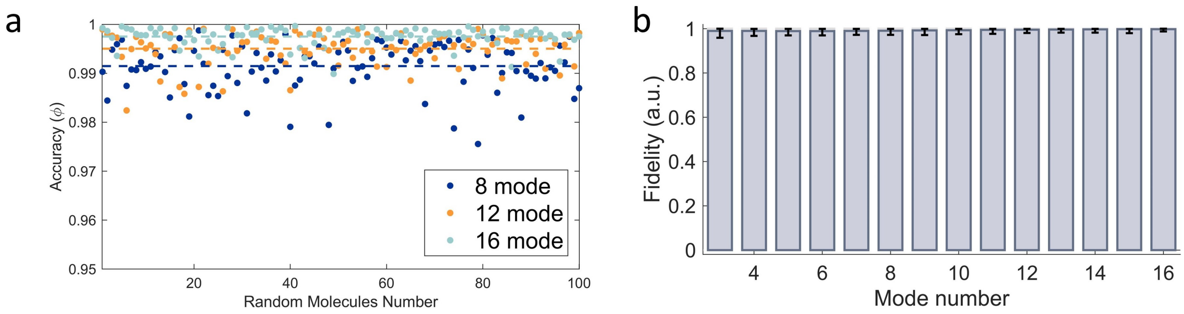

Supplementary Figure 2: **a** Fidelity of vibronic spectra distribution for all random virtual molecules (dots). Dashed lines are the mean values of fidelity per mode size. **b** Summary of statistical fidelity for different molecular mode sizes.

Supplementary Note 2: Complexity Analysis

In this section, we discuss the complexity of obtaining molecular vibronic spectra in three aspects: classical computation, quantum simulation based on squeezed coherent states, and our new method based on squeezed vacuum states. Calculating the molecular vibronic spectra with classical algorithms seems challenging due to two reasons. (a) The FCF associated with a transition between initial and final vibrational states in two different potential energy surfaces is equivalent to calculating the number of perfect matchings of a weighted graph with loops [6], which is known as the loop hafnian. Computing the loop hafnian is #P-hard, and the renowned classical algorithm, specifically the eigenvalue-trace algorithm, exhibits a complexity of $O(N^3 2^{(N/2)})$ with N being the matrix size [12]. (b) The density of vibrational states increases dramatically with the number of atoms and internal energy. Therefore, obtaining the entire FCP requires a large number of results, making the molecular vibration spectrum problem exponentially harder in molecular size. Then, a boson sampling device is applied to solve the molecular vibronic spectra in quantum chemistry [10]. However, there is a concern that the histogram of boson sampling estimation can also be estimated using several polynomial-time classical algorithms [13–15]. Recently, Ref. [16] proposed a quantum-inspired classical algorithm, the generalized Gurvits's algorithm, and analyzed the applicable and inapplicable conditions of this classical algorithm. The study reveals that the generalized Gurvits's algorithm can be adapted to estimate the molecular vibronic spectra corresponding to the Fock-state or Gaussian boson sampling with large squeezing [16]. However, no known efficient classical algorithm can approximate the general case whereby the initial state is the Fock state with squeezing and non-zero displacement. As a result, the quantum-vs-classical separation in the computational complexity of the molecular vibronic spectra problem is not completely determined yet. Thus, exploring the molecular vibronic spectra problem using quantum methods is crucial, particularly considering its potential for providing a quantum advantage for molecules that cannot be simulated using

classical algorithms.

The relationship between the Doktorov operator describing the transformation of vibronic state and the operators associated with displacement, squeezing and rotation in quantum optics is tactfully established, proposing a modification of boson sampling for the purpose of quantum simulation [10]. This approach enables us to convert this computationally challenging problem into a sampling task, allowing for the straightforward estimation of spectra at each given vibrational transition frequency (ω_{vib}) by collecting the output patterns. We need to declare that the number of samples required to reach a desired precision (ϵ) scales as $O(\epsilon^{-2})$. It means that a polynomial number of samples is sufficient to estimate the FCP if the goal is to construct an overall profile with reasonable precision [10].

We construct the relationship between the FCF with a weighted summation of the hafnian function, which corresponds to the probability distribution of a boson sampling problem with a squeezed vacuum state in **Supplementary Note 1**. In addition, doubling the original modes ensures that each sample maps to a single transition frequency and that the number of post-summations will not be greater than the number of samples. The detailed derivation of the scalability of the sampling process is shown in **Methods**. Furthermore, the numerical simulation is used to evaluate the number of summation terms. The relationship between the number of summation terms, denoted as t, and the molecular size is shown in **Supplementary Figure 3**, where the molecular size ranging from 2 to 15 are simulated. It can be seen that the probability of occurrence of many combinations of samples is extremely low, the average number of summation terms is lower than 2 and the maximum t does not increase with the molecular size. Thus, the sum operation does not add a lot of computational burdens. In **Supplementary Figure 3**, the fidelity between the theory and simulation versus molecular size with 10000 samplings is calculated. It is further verified from the simulation aspect that our method is sufficient to estimate the FCP of molecules.

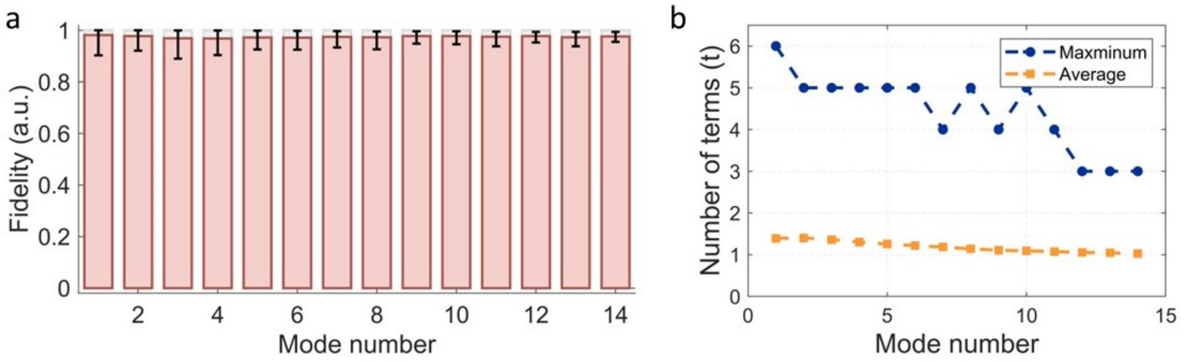

Supplementary Figure 3: **a** Fidelity between theory and simulation versus selected mode number. The error bar represents 95% confidence interval. **b** Number of summation terms in the sampling process versus the selected mode number.

In summary, when addressing the challenging task of simulating molecular vibronic spectra, the eigenvalue-trace algorithm exhibits an exponential runtime in terms of the molecular size, and an existing quantum algorithm (a boson sampler with displacements) is fraught with experimental difficulties. As an alternative quantum approach, we propose to employ a boson sampler without displacements that offer the ease of implementation.

Supplementary Note 3: Experimental details

A. Photon source

There are two schemes to produce photons: (1) in the single-wavelength pumping scheme, signal and idler photons are generated at different wavelengths, and (2) in the dual-wavelength pumping scheme, photons are generated at the same signal wavelength. For our boson sampling protocol, the dual-wavelength pumping scheme is chosen in the experiment. The photonic states used as inputs for the simulations are generated using a dual-pump pulse light produced by the Ultrafast Optical Clock device (PriTel) with a repetition rate of 500 MHz, a central wavelength of 1550.116 nm, and a bandwidth of 1.9 nm. The two pulse light with wavelengths of 1546.8 nm and 1553.2 nm in our experiment is selected through a compressor (PriTel) to expand the bandwidth to about 10 nm, followed by a 100G Wavelength Division Multiplexer (WDM) device. To increase the input light power for increasing the squeezing level of a squeezed vacuum state, an Erbium dope fiber amplifier (EDFA) and another pair of WDM are employed to amplify the input light and suppress the light power at wavelengths other than the two selected ones, respectively. The optical paths of the two pump wavelengths are balanced by connecting a tunable delay line to one arm of the channel to overlap the two pulses. The dual pump light is launched into the device through a subwavelength grating coupler, and the polarization controller maintains the light at the maximal TE polarization. Up to sixteen pairs of a single-mode squeezed vacuum state can be generated by the spontaneous four-wave mixing (SFWM) process from the sixteen spiral lines (a single-pass squeezer) on the integrated photonic chip.

B. Integrated photonic chip

The chip is fabricated on a commercial silicon-on-insulator (SOI) wafer with a top silicon thickness of 250 nm and a buried oxide layer of 3 μm. The waveguide structure is patterned by photolithography, prior to inductively coupled plasma (ICP) etching of the silicon layers. Then, a 3-μm-thick SiO_2 layer is deposited via plasma-enhanced chemical vapor deposition (PECVD) to form the upper cladding. Subsequently, a 150-nm-thick TiN thin film is deposited by electron beam evaporation (EBE) and patterned as heaters. After a successive SiO_2 PECVD process, the aluminum electrodes are deposited and patterned to connect the heaters and electrode pads. Then, a thin aluminum oxide Al_2O_3 layer is deposited on the top of the chip for electrical insulation. Finally, the deep reactive

ion etching (DRIE) is used to create the deep trench and cutting line of the chip.

Once the device is fabricated, the basic components on the chip are first characterized. The waveguides on our device are fully-etched silicon waveguides with a cross-section of 500×250 nm. The propagation loss through straight waveguides was estimated via cut-back measurements, which is approximately 1.5 dB cm^{-1}. To reduce the coupling loss, the edge coupling with a taper is used to couple photons out of the chip and has an efficiency of around -1.5 dB for the wavelength of 1550.116 nm. For the boson sampling circuit, the core is the 16-mode universal interferometer network, which consists of 120 MZIs and 240 phase shifters to perform any unitary transformation of the optical modes using the theoretical scheme described in Ref [17]. Each of the thermal phase shifters consists of a titanium electrical heater to change the temperature, and, thereby, the refractive index of the silicon waveguides, powered by an electronic circuit externally controlled by a computer. The average resistance of the phase shifters on the device is around $500\,\Omega$ and access a 2π phase shift within the current range $[0, 3]$ mA. For the source, the spiral line composed of single-mode waveguides (2 cm length) to produce a squeezed vacuum state, and asymmetric MZIs (AMZIs) with arm-length difference $\Delta L = 90\,\mu$m is used to route the pump light and produce photon frequencies to different output ports of the AMZIs.

The above components contribute to the overall quantum photonic microprocessor chip. The 13×4 mm^2–sized chip monolithically integrates 16 squeezing sources, 272 thermo-optic phase shifters, 319 multimode interferometer beam splitters, and 65 optical grating couplers.

C. Detectors and Probabilistic photon number resolving

The output photons are first filtered by sixteen off-chip filters (1.2 nm bandwidth, 0.75 dB average insertion loss) to remove spurious pump photons and enhance the photon indistinguishability, and measured by an array of single photon detectors. The 16 channels of fiber-coupled superconducting nanowire single-photon detectors (SNSPDs) (Photon SoptTM) with 100 Hz dark counts and an average efficiency of 85% are used to detect photons. A time tagger (Swabian Instrument TM GmbH) is connected to the SNSPDs to obtain coincidence counting values. The time window in the experiment is usually set to be around 800 ps. The time delay of each channel is set through the counting card in the time tagger to compensate for the discrepancy in the signal's arrival time introduced in the experiment by optical fibers, detectors, electronics, and coaxial cables.

Photon-number-resolving detectors are needed in our experiment of molecular vibrational simulation. Path-multiplexing techniques are used to implement pseudo number resolving photo-detection, allowing the threshold detectors (Photon SoptTM) to be used as photon-number-resolving detectors. This is achieved by inserting beam splitters at each relevant mode and connecting the split multi-ports to the detectors. For a beam splitter with reflectivity R_f connected to the detectors with efficiencies η_1 and η_2, the probability of detecting a coincidental detection in a two-photon input is $2R_f(1-R_f)_{12}$. After normalizing the detector's efficiencies and setting $R_f =$

$1/2$, the coincidental count rate of two output modes is half of the input two-photon counts. For more complex splitting situations using three beam-splitters to split one mode to four output modes, the probability relation between the input photon numbers and the output coincidence photon numbers is detailed in **Table 1**. The threshold detectors can be used as photon-number detectors by re-normalizing all coincidence counts based on the probability distribution in **Table 1**.

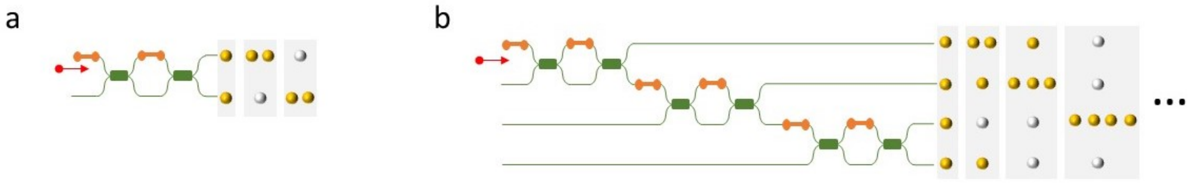

Supplementary Figure 4: Detection schematic for pseudo-photon number resolving measurements. **a** two-photon resolving. **b** four-photon resolving.

Table **1**: The probability distribution for pseudo-photon number resolving measurements.

	4 photons coincidence	3 photons coincidence	2 photons coincidence	1 photons coincidence
4 photons	3/32	9/16	21/64	1/64
3 photons	0	3/8	9/16	1/16
2 photons	0	0	3/4	1/4

D. Electrical and thermal packaging

For the electrical packaging, high-density (two-layer) wire-bonding technology is used to connect the electrical pads on the chip to the PCB pads. Phase shifters on the device are configured through a digital-to-analog converter (Qontrol Systems) with 16-bit and 305 μV resolution and controlled by the computer. A Peltier controlled by Thorlabs TED200C and a water-cooling substrate under the chip are used to control and stabilize the temperature using a temperature controller. The added cooling system further reduces the heat fluctuations caused by ambient temperature and the heat crosstalk within the chip.

Supplementary Note 4: Device performance

A. Source characterization

To pump the on-chip sources, laser pulses are first generated and the AMZI with free spectral range (FSR) of 6.32 nm is used to filter the generated squeezed state from the spiral lines. In our experiment, we modulate the temporal delay to obtain the maximum photons emitted by the degenerate SFWM. Once the device's active components are characterized, preliminary tests on the squeezing sources can be performed. When one of the sources is connected directly to the detectors, the calculated squeezing value by second-order correlation measurements reaches around 0.3 (2.6 dB) when the input power is around 0.38 mW (**Supplementary Figure 5a**). The count value of photons is around 4 kHz (**Supplementary Figure 5b**). The measured average purity of 16 TMSSs is around 0.7 (**Supplementary Figure 5c**). The calculated ratio between the coincidence detection rate and the single detection rate is 2% when the on-chip source goes through the 16-mode network to the detectors. Taking into account of the detector efficiency, waveguide loss, and coupling loss, this translates to a heralding efficiency of around 80%.

The source emission's single-mode squeezing (SMS) parameter is calculated using two-mode second-order correlation measurements in the experiment setup schematized in Figure 2. Ref. [18] reported the calculation of the two-mode squeezing (TMS) parameter of the source using two-mode second-order correlation measurements. Our method is similar to this by changing the source state to SMS state instead of TMS. Given a pure single-mode SMS state, the probability of detecting $n_s(n_i)$ single photons in the signal (idler) mode in the lossless case is expressed as

$$p_{SMS} = (n_s n_i) = \frac{\tanh^{2n_s}(\xi)(2n_s)!}{\cosh(\xi)2^{2n_s}n_s!n_s!}\delta_{n_i,n_s} = p_0(n_s)\delta_{n_i,n_s}. \tag{30}$$

In the presence of losses, the detection probability is given by

$$
\begin{aligned}
p\left(n_s,n_i\right) &= \left(\frac{\eta}{1-\eta}\right)^{n_s+n_i} \sum_{n\geq \max\left\{n_s,n_i\right\}} p_0\left(n\right)\left(1-\eta\right)^{2n} \\
&= A\left(\xi,\eta\right)\left(\frac{\eta}{1-\eta}\right)^{n_s+n_i} \sum_{n\geq \max\left\{n_s,n_i\right\}} \frac{\tan\mathrm{h}^{2n}\left(\xi\right)\left(2n\right)!}{2^{2n}n!n!}\left(1-\eta\right)^{2n} \\
&= A\left(\xi,\eta\right)\left(\frac{\eta}{1-\eta}\right)^{n_s+n_i} \sum_{n\geq \max\left\{n_s,n_i\right\}} \tan\mathrm{h}^{2n}\left(\xi\right)\frac{1}{\sqrt{\pi n}}\left(1-\eta\right)^{2n} \\
&= A\left(\xi,\eta\right)\left(\frac{\eta}{1-\eta}\right)^{n_s+n_i} \sum_{n\geq \max\left\{n_s,n_i\right\}} \left(a_1b_1^{2n}+a_2b_2^{2n}+a_3b_3^{2n}\right)tanh^{2n}\left(\xi\right)\left(1-\eta\right)^{2n} \\
&= A\left(\xi,\eta\right)\left(\frac{\eta}{1-\eta}\right)^{n_s+n_i}\left\{\begin{array}{l} a_1\left(\sum_{n=0}^{\infty}\left[b_1\tan\mathrm{h}\left(\xi\right)\left(1-\eta\right)\right]^{2n}-\sum_{n=0}^{\max(n_s,n_i)}\left[b_1\tan\mathrm{h}\left(\xi\right)\left(1-\eta\right)\right]^{2n}\right) \\ +a_2\left(\sum_{n=0}^{\infty}\left[b_2\tan\mathrm{h}\left(\xi\right)\left(1-\eta\right)\right]^{2n}-\sum_{n=0}^{\max(n_s,n_i)}\left[b_2\tan\mathrm{h}\left(\xi\right)\left(1-\eta\right)\right]^{2n}\right) \\ +a_3\left(\sum_{n=0}^{\infty}\left[b_3\tan\mathrm{h}\left(\xi\right)\left(1-\eta\right)\right]^{2n}-\sum_{n=0}^{\max(n_s,n_i)}\left[b_3\tan\mathrm{h}\left(\xi\right)\left(1-\eta\right)\right]^{2n}\right)\end{array}\right\} \\
&= B\left(\xi,\eta\right)\left(\frac{\eta}{1-\eta}\right)^{n_s+n_i}\left\{\frac{a_1}{q_1\left(\xi,\eta\right)}\left[b_1tanh\left(\xi\right)\left(1-\eta\right)\right]^{2\max n_s,n_i}\right. \\
&\left.+\frac{a_2}{q_2\left(\xi,\eta\right)}\left[b_2tanh\left(\xi\right)\left(1-\eta\right)\right]^{2\max n_s,n_i}+\frac{a_3}{q_3\left(\xi,\eta\right)}\left[b_3tanh\left(\xi\right)\left(1-\eta\right)\right]^{2\max n_s,n_i}\right\},
\end{aligned} \tag{31}
$$

where $a_k, b_k = 0.5913,\ \ 0.4883; 0.2705,\ \ 0.9243; 0.1357,\ \ 0.995$, $q_i = 1 - b_i\ \tanh(\xi)\ (1-\eta)\ (i = 1,\ 2,\ 3)$ and $A(\xi,\eta)$ and $B(\xi,\eta)$ are the normalization constants.

To determine the constant $B(\xi,\eta)$, the normalization condition $\sum_{(n_s,n_i=0)}^{lim} p(n_s,n_i\ ) = 1$ is imposed and rewritten as

$$
\sum_{n_s,n_i=0}^{lim} p\left(n_s,n_i\right) = 1 = 2\sum_{n_i=0}^{lim}\sum_{n_s\geq n_i} p\left(n_s,n_i\right) - \sum_{n_i=0}^{lim} p\left(n,n\right), \tag{32}
$$

and

$$
\frac{a_1B\left(\xi,\eta\right)}{\xi\left(a\right)\left(1-\eta^2b_1^2tanh^2\xi\right)q_1\left(\xi,\eta\right)}+\frac{a_2B\left(\xi,\eta\right)}{\xi\left(b\right)\left(1-\eta^2b_2^2tanh^2\xi\right)q_2\left(\xi,\eta\right)}+\frac{a_3B\left(\xi,\eta\right)}{\xi\left(c\right)\left(1-\eta^2b_3^2tanh^2\xi\right)q_3\left(\xi,\eta\right)}=1, \tag{33}
$$

where $a = \eta(1-\eta)tanh^2\xi b_1^2$, $b = \eta(1-\eta)tanh^2\xi b_2^2$, $c = \eta(1-\eta)tanh^2\xi b_3^2$ and $\xi(x) = (1-x)/(1+x)$.

Then, the correlation measurements at a time delay $\Delta t = 0$ will thus represent events whenever $n_s \geq 1$ signal photons and $n_i \geq 1$ idler photons from a single SMS arrive at the detectors simultaneously, which happens with a probability

$$
\begin{aligned}
p_{coinc}\left(\Delta t=0\right) &= p\left(n_s\geq 1, n_i\geq 1\right) \\
&= 1-p\left(0,\ 0\right)-p\left(0,\ 1\right)-p\left(1,\ 0\right) \\
&= 1-\left(\frac{a_1}{q_1}+\frac{a_2}{q_2}+\frac{a_3}{q_3}\right)B-2\left(\frac{a_1b_1^2}{q_1}+\frac{a_2b_2^2}{q_2}+\frac{a_3b_3^2}{q_3}\right)Btanh^2\left(\xi\right)\eta\left(1-\eta\right).
\end{aligned} \tag{34}
$$

The probability of having this event with a time delay $\Delta t > 0$ is

$$\begin{aligned} p_{coinc}(\Delta t > 0) &= p(n_s \geq 1, n_i \geq 1) \\ &= \left(\sum_{n_i=0}^{lim} p(n_s \geq 1, n_i)\right)\left(\sum_{n_s=0}^{lim} p(n_s, n_i \geq 1)\right) \\ &= (1 - \frac{a_1 B}{q_1(1-a)} - \frac{a_2 B}{q_2(1-b)} - \frac{a_3 B}{q_3(1-c)})^2. \end{aligned} \tag{35}$$

Then, the quantity to be considered is the ratio between the coincidences measured at $\Delta t > 0$ and those measured at $\Delta t = 0$,

$$R = \frac{p_{coinc}(\Delta t > 0)}{p_{coinc}(\Delta t = 0)}. \tag{36}$$

In our experiment, the squeezing parameters are set low and losses in the detection channels are significant. Under this approximation, an estimation equation of the squeezing parameter is obtained via

$$tanh^2 \xi \approx \frac{2R}{1 - 2R}. \tag{37}$$

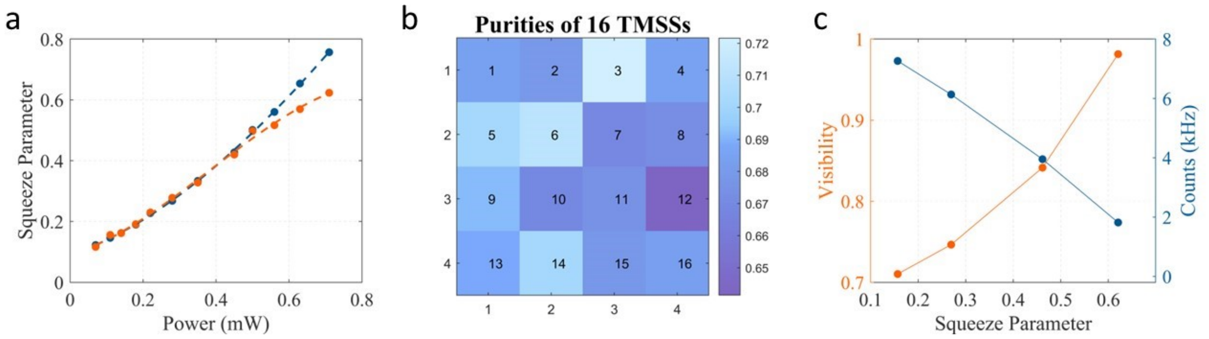

Supplementary Figure 5: Characterization of degenerate SFWM squeezing source. **a** Squeezing parameters are measured over the power increase. **b** Purity of the 16 photon sources obtained by unheralded second-order correlation measurement. **c** Visibility and photon pair event rates are measured over the squeezing parameters increase.

B. Matrix characterization

Given the large-scale matrix operation in our experiment, we also perform some matrix reconstruction experiments and chip performance validation. Phase shifters in our design are used to modulate the matrices composed of

AMZIs and MZIs. The calibration of one phase shifter is shown in **Supplementary Figure 6a** and the histograms of the visibilities for all 318 phase shifters in the circuit are depicted in **Supplementary Figure 6b**. The phase θ is controlled by a heater and, when the power is switched on, the heater induces refractive index change by thermal effect and causes the phase difference between the two arms. We have prechecked the resistance of the heater to be a constant such that the relation between phase θ and power I^2 is linear and the final form for the output light intensity can be written as $P = V_0 + V_0 k cos^2(\omega I^2 + \theta_0)$. The measured interference pattern shows the periodical change by the increase of I^2 (**Supplementary Figure 6a**). The visibility k of the interference fringe is defined as $k = \frac{Pmax-Pmin}{Pmax+Pmin}$. From **Supplementary Figure 6b**, 96% of the heaters possess a visibility over 95%.

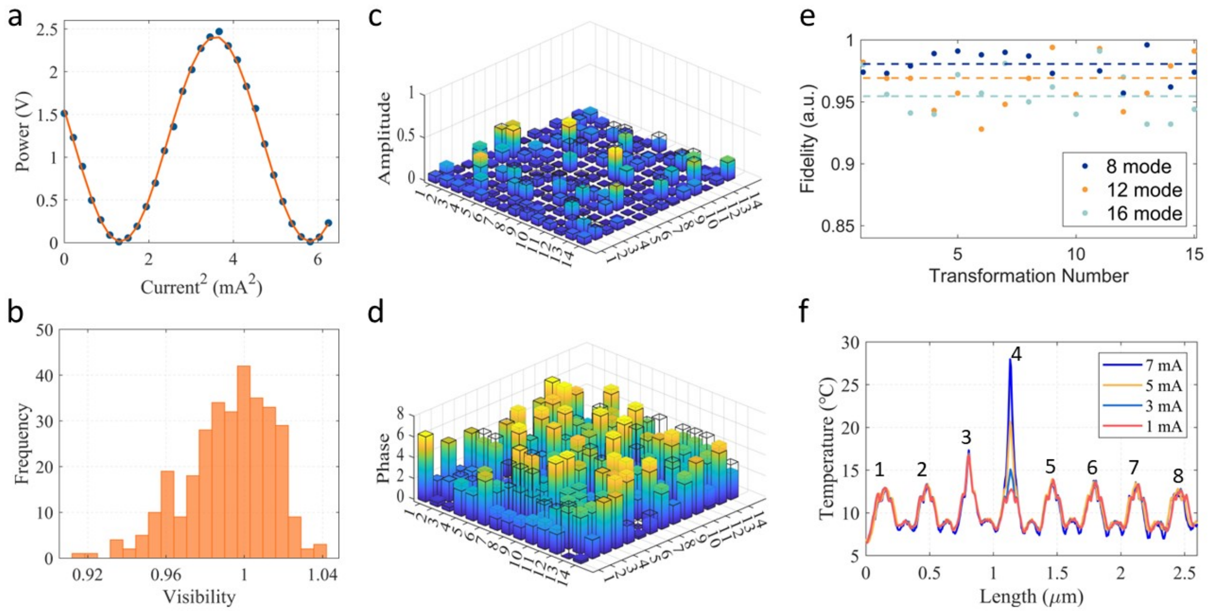

Supplementary Figure 6: Calibration of the matrix. **a** and **b** Calibration of phase shifters. **c** and **d** Reconfigured amplitudes and phases of one random 14 modes complex unitary matrices. **e** Fidelity distribution of the target unitary and the measured one for all transformations (dots). **f** Monitored temperature change under different currents of the heater.

The network scheme in our experiment is a square circuit [17] and an arbitrary 16-mode unitary matrix can be decomposed into parameters of each MZI unit (θ, ϕ). The transfer function of one MZI unit is

$$T = ie^{i\theta} \begin{bmatrix} e^{i\phi} \sin\left(\frac{\theta}{2}\right) & \cos\left(\frac{\theta}{2}\right) \\ e^{i\phi} \cos\left(\frac{\theta}{2}\right) & -\sin\left(\frac{\theta}{2}\right) \end{bmatrix}, \tag{38}$$

where θ is an inner phase of MZI and ϕ is an outer phase of MZI. After that, 60 random matrices within the

interferometer are generated and implemented on the chip. The constructed corresponding amplitudes and phases of the random unitary matrix with 14×14 modes are shown in **Supplementary Figure 6c** and **Supplementary Figure 6d**, respectively, in which color and transparent bars represent the experimental and theoretical results, respectively. A fidelity distribution of the 15 arbitrary unitary transformations per investigated matrix dimension (different colors belong to different dimensions) is displayed in **Supplementary Figure 6e**. Though the average fidelity (dashed line) decreases with the increasing dimension of the transformation, the measured average fidelities are always higher than 0.95. We further measure the temperature variation across the whole chip (**Supplementary Figure 6f**) when only one heater's current increases and observe that the thermal crosstalk of individual tunable elements remains below $1°$C. This further verifies the excellent matrix reconstruction from another aspect.

C. Quantum characterization

We consider the quantum characterization by computing the statistical results on the acquired event data for different interferometer configurations and investigating the average fidelity of two-photon and four-photon clicks in the output (**Supplementary Figures 7a** and **7b**). The measured fidelity in the experiment of calculating formic acid molecule's vibration spectrum with two-photon clicks is 94.5% and that with four-photon clicks is 92.0%. The concluded measured average fidelity in our experiment of two-photon and four-photon clicks in the output is shown in **Supplementary Figure 7c** and they are always higher than 90% for two-photon clicks and higher than 80% for four-photon clicks. **Supplementary Figure 7d** depicts typical examples of the Bayesian model comparison for 4-photon clicks [19], showing a stark difference between squeezing states with thermal and uniform states.

Supplementary Note 5: Molecule details

Here, we list all computed characteristic parameters including vibrational frequencies and transformation matrices used in the work. As defined in **Supplementary Note 1**, U represents the Duschinsky matrix, ω and ω' denote the harmonic angular frequencies of the final and initial states, and **K** signifies the displacement vector responsible for the molecular structural changes along the normal coordinates. The vibrational frequencies are in cm^{-1}, while the other quantities are dimensionless. The vibronic transition parameters of formic acid and thymine are obtained from Ref. [5], of naphthalene, phenanthrene, and benzene are obtained from Ref. [20] and of pyrrole are obtained from Ref. [21].

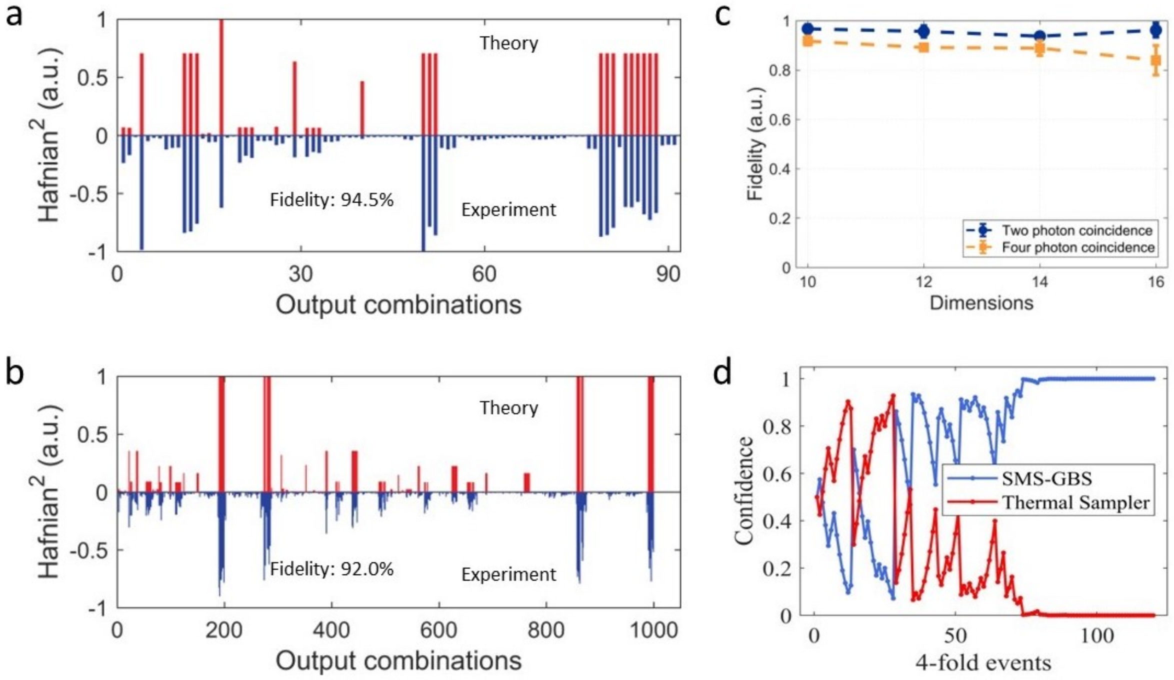

Supplementary Figure 7: Quantum characterization of the microprocessor. **a** and **b** Experimental (red) and theoretical (blue) two and four-photon distribution for Formid acid molecule. **c** Summary of statistical fidelities of two-photon and four-photon distributions for 4 different matrix dimensions. **d** Validation against thermal state and uniform state hypothesizes with detected photon number of four photons.

A. Formic Acid

$$U = \begin{pmatrix} 0.8423 & 0.1799 & -0.3857 & 0.3074 \\ -0.3403 & -0.5231 & -0.6679 & 0.3848 \\ -0.4004 & 0.7636 & -0.1036 & 0.4838 \\ -0.0907 & 0.3151 & -0.5900 & -0.7193 \end{pmatrix},$$

$$\omega = \begin{pmatrix} 1825.1799 \\ 1416.9512 \\ 1326.4684 \\ 1137.04 \end{pmatrix}, \omega' = \begin{pmatrix} 1566.4602 \\ 1399.6554 \\ 1215.3421 \\ 1190.9077 \end{pmatrix}, \mathbf{K} = \begin{pmatrix} 1.5599 \\ -0.3784 \\ 0.4553 \\ -0.3439 \end{pmatrix}.$$

B. Thymine

$$U = \begin{pmatrix} -0.0373 & 0 & 0 & 0 & 0 & 0 & 0 \\ 0 & -0.3441 & 0.0318 & -0.2303 & -0.1322 & 0.162 & 0.1761 \\ 0 & 0.2715 & -0.1283 & 0.2791 & 0.4629 & 0.0143 & 0.2017 \\ 0 & 0.2184 & -0.262 & 0.2368 & -0.2354 & 0.0205 & -0.1328 \\ 0 & 0.0153 & -0.2638 & -0.1924 & -0.1226 & 0.0199 & -0.1291 \\ 0 & -0.1801 & 0.1629 & 0.1492 & -0.063 & -0.0567 & 0.4707 \\ 0 & -0.1157 & -0.1349 & 0.1014 & -0.1531 & 0.1296 & 0.0048 \end{pmatrix},$$

$$\omega = \begin{pmatrix} 108.354 \\ 390.234 \\ 547.949 \\ 732.620 \\ 1219.420 \\ 1372.674 \\ 1697.986 \end{pmatrix}, \omega^{'} = \begin{pmatrix} 57.567 \\ 392.319 \\ 532.098 \\ 690.284 \\ 1270.82 \\ 1314.60 \\ 1574.93 \end{pmatrix}, \mathbf{K} = \begin{pmatrix} 0 \\ -0.7399 \\ -0.6850 \\ 0.6530 \\ 0.4817 \\ 0.8490 \\ -0.8732 \end{pmatrix}.$$

C. Naphthalene

$$U = \begin{pmatrix} 0.9798 & -0.2 \\ 0.2 & 0.9798 \end{pmatrix},$$

$$\omega = \begin{pmatrix} 509 \\ 938 \end{pmatrix}, \omega^{'} = \begin{pmatrix} 438 \\ 912 \end{pmatrix}, \mathbf{K} = \begin{pmatrix} 0 \\ 0 \end{pmatrix}, \mu = \begin{pmatrix} 1 \\ -1 \end{pmatrix}.$$

D. Phenanthrene

$$U = \begin{pmatrix} 0.9055 & -0.424 \\ 0.424 & 0.9055 \end{pmatrix},$$

$$\omega = \begin{pmatrix} 700 \\ 800 \end{pmatrix}, \omega^{'} = \begin{pmatrix} 679 \\ 796 \end{pmatrix}, \mathbf{K} = \begin{pmatrix} 0.165 \\ 0.078 \end{pmatrix}, \mu = \begin{pmatrix} 1.5 \\ -0.5 \end{pmatrix}.$$

E. Benzene

$$U=\begin{pmatrix} 0 & 1 & 0 & -0.0013 & 0 & -0.0009 & -0.0019 & 0 \\ 1 & 0 & 0.0013 & 0 & 0.0009 & 0 & 0 & 0.0019 \\ -0.0012 & 0 & 0.9988 & 0 & -0.0485 & 0 & 0 & -0.003 \\ 0 & 0.0012 & 0 & 0.9988 & 0 & -0.0485 & -0.003 & 0 \\ -0.0009 & 0 & 0.0485 & 0 & 0.9988 & -0.0001 & 0 & 0.0092 \\ 0 & 0.001 & 0 & 0.0486 & 0.0001 & 0.9988 & 0.0092 & 0 \\ -0.0019 & 0 & 0.0025 & 0 & -0.0094 & 0 & 0 & 1 \\ 0 & 0.0019 & 0 & 0.0025 & 0 & -0.0094 & 1 & 0 \end{pmatrix},$$

$$\omega=\begin{pmatrix} 3369.222 \\ 3369.222 \\ 1730.1965 \\ 1730.1964 \\ 1263.3599 \\ 1263.3599 \\ 646.1398 \\ 646.1398 \end{pmatrix}, \omega^{'}=\begin{pmatrix} 3389.0368 \\ 3389.0368 \\ 1665.3003 \\ 1665.3003 \\ 1236.7103 \\ 1236.7103 \\ 575.1367 \\ 575.1367 \end{pmatrix}, \mathbf{K}=\begin{pmatrix} 0 \\ 0 \\ 0 \\ 0 \\ 0 \\ 0 \\ 0 \\ 0 \end{pmatrix}, \mu=\begin{pmatrix} 0.3054 & 0 \\ 0 & 0.3054 \\ 0 & -0.1795 \\ 0.1795 & 0 \\ 0.119 & 0 \\ 0 & -0.119 \\ 0 & -0.5710 \\ 0.5710 & 0 \end{pmatrix}.$$

F. Pyrrole

$$U=\begin{pmatrix} 0.4537 & 0.5603 & 0.2319 & -0.0495 & -0.5757 & -0.0153 \\ -0.4367 & 0.1205 & 0.0304 & 0.0188 & -0.1154 & 0 \\ -0.1326 & 0.227 & 0.2054 & -0.1956 & 0.0574 & -0.0931 \\ 0.0223 & -0.0033 & -0.0024 & -0.0004 & 0.0117 & -0.0046 \\ -0.0016 & -0.0003 & -0.001 & -0.0008 & 0.001 & 0.6067 \\ 0.0567 & 0.1814 & 0.623 & 0.5296 & 0.5074 & -0.0033 \end{pmatrix},$$

$$\omega=\begin{pmatrix} 905.61 \\ 1117.26 \\ 1191.51 \\ 1462.23 \\ 1538.1 \\ 1615.27 \end{pmatrix}, \omega^{'}=\begin{pmatrix} 882.31 \\ 1215.65 \\ 1080.48 \\ 1589.65 \\ 1518.11 \\ 1492.94 \end{pmatrix}, \mathbf{K}=\begin{pmatrix} -0.511 \\ 0.2628 \\ -0.7884 \\ -0.1865 \\ -0.7961 \\ 0.8472 \end{pmatrix}.$$

Supplementary Note 6: Data encoding details

We reconstruct the FCP for the synthetic molecule directly associated with the device, $i.e.$, with a vibronic transition described by the squeezing parameters **r** and the unitary V implemented in our device. The unitary V can be easily performed through the interferometer network, but, in our experiment, we can only access a limited amount of squeezing. Therefore, for each source, we can only implement a squeezing $r_i \leq r_{max}$. Furthermore, the relative values of the squeezing sequence also need to keep close for easier implementation experimentally. Thus, we further rewrite the parameter of matrix $A^{'}$,

$$
\begin{aligned}
C &= \mathrm{diag}\left(w_1, w_2\right) A^{'} \mathrm{diag}\left(w_1, w_2\right) \\
&= \begin{bmatrix} w_1^2 B & w_1 w_2 Z \\ w_1 w_2 Z & 0 \end{bmatrix}.
\end{aligned} \tag{39}
$$

Then, Eq. (28) can be rewritten as

$$
\mathrm{FCF}\left(\mathbf{m}, \mathbf{n}\right) = \mathcal{N} |\sigma_Q|^{\frac{1}{4}} w_1^{-sum(\mathbf{p})} \sum_{\mathbf{n}=0}^{\mathbf{p}} w_2^{-sum(\mathbf{l})} \left(\frac{Pr\left(\mathbf{p}, \mathbf{l}\right)}{\mathbf{l}!} \right)^{1/2}, \tag{40}
$$

where $0 < w_1, w_2 \leq 1$ with a real constant rescaling weight. Considering uniform losses η in the device and the pseudo-photon number resolving probability p, we have a relation between the ideal FCFs $p_{FC}(\mathbf{k})$ and the ones reconstructed in the imperfect device $\bar{p}_{FC}(\mathbf{k})$, given by

$$
\bar{p}_{FC}\left(\mathbf{k}\right) = \mathcal{N} \eta^n p\left(\mathbf{k}\right) p_{FC}\left(\mathbf{k}\right), \tag{41}
$$

where $n = \sum_{i=1}^{m} k_i$ is the total number of photons in the output configuration **k**, $\mathcal{N}$ a normalization constant, and the extra probability $p(\mathbf{k})$ arising from pseudo-photon number resolving detection. Then, Eq. (41) can be inverted to reconstruct the ideal FCP.

Supplementary Note 7: Error and scalability analysis

In our algorithm to calculate the vibration spectrum, the size of the mode number and detected photon numbers make the resulting spectrum congested because of the increase in the density of states. Therefore, the core of scaling is increasing the photon and mode numbers. It should be noted that performing data post-processing does not increase the computational complexity of the experiment. However, it is important to consider that the FCP is truncated at n-photon contributions when we collect only up to n-photon events. Consequently, the reconstructed FCP is accurate only when the ideal contributions involving more than n photons are insignificant. This condition holds true for high values of n only when the efficiency η is close to unity.

One method to increase efficiency is to reduce the optical losses, in particular, the insertion loss of integrated optical components and the coupling loss. With the propagation loss through straight waveguides in our experiment, it is to be approximately 1.5 dB cm^{-1}, the transmission loss for the 16-mode boson sampling circuit is 2 dB. Similarly, the losses for the MMI are around 0.15 dB. The spiral source experiences higher propagation loss due to bending losses, which on average 4.5 dB over a 1.4 cm long spiral. To minimize coupling loss, we use a high-efficiency optical fiber to edge-couple the output waveguide, which results in a lower loss of 1.5 dB. Finally, considering the detector is a polarization-sensitive device, the off-chip filters and the polarization controller are added before the detectors. The measured average transmission efficiencies over the 16 channels are 0.6 dB and 0.1 dB, respectively.

Enhancing the performance of the microprocessor and extending the chip size to simulate more complex molecules can be achieved by reducing the noise of the chip. The core noise in our chip is primarily caused by thermal crosstalk and inaccurate phase value settings, leading to decreased fidelity for larger networks. Maintaining a high-accuracy network is crucial in reducing such noise, with the boson sampling network consisting of programmable MZIs modulated by thermal heaters. Heat dissipation significantly affects the accuracy of the setting network, making thermal management a critical issue in further integrating and miniaturizing optical components. To reduce heat dissipation from the TiN heater to the Si substrate and the surrounding SiO_2, thermal isolation trenches are added to the chip. Additionally, an external water-cooling system and temperature controller are included to decrease heating crosstalk and maintain chip temperature stability. The accumulated heat throughout the whole chip cannot be ignored when the network size is very large, leading to phase calibration curve drift. While we still have many steps to take toward future large-scale implementations, near-term quantum devices benefit significantly from our current progress.

Besides enhancing the chip performance, photon loss calibration and increased photon number resolution can also reduce errors. Within the theoretical framework, one-photon events are defined as a_i (where i denotes the mode label), two-photon events as $b_{(i,j)}$ (with i and j as mode labels), three-photon events as $c_{(i,j,k)}$ (with i, j, and k as mode labels), and four-photon events as $d_{(i,j,k,l)}$ (with i, j, k, and l as mode labels). To account for photon loss, we introduce a loss parameter η. Consequently, the experimentally obtained one-photon event, denoted as a'_i, can be expressed as

$$a'_i = (1-\eta)a_i + \eta(1-\eta)\sum_j b_{(i,j)} + \eta^2(1-\eta)\sum_{(j,k)} c_{(i,j,k)} + \eta^3(1-\eta)\sum_{(j,k,l)} d_{(i,j,k,l)+\cdots}. \tag{42}$$

Similarly, we can establish the relationship between the experimental and theoretical two-photon, three-photon, and four-photon events, and so forth. This equation demonstrates that experimentally obtained one-photon events encompass not only the primary one-photon events but also the components lost from multi-photon events. After correction, this source of error can be mitigated. Furthermore, an increase in the resolution of the photon number

counting can reduce the likelihood of erroneously classifying a multiphoton event as a single-photon event.

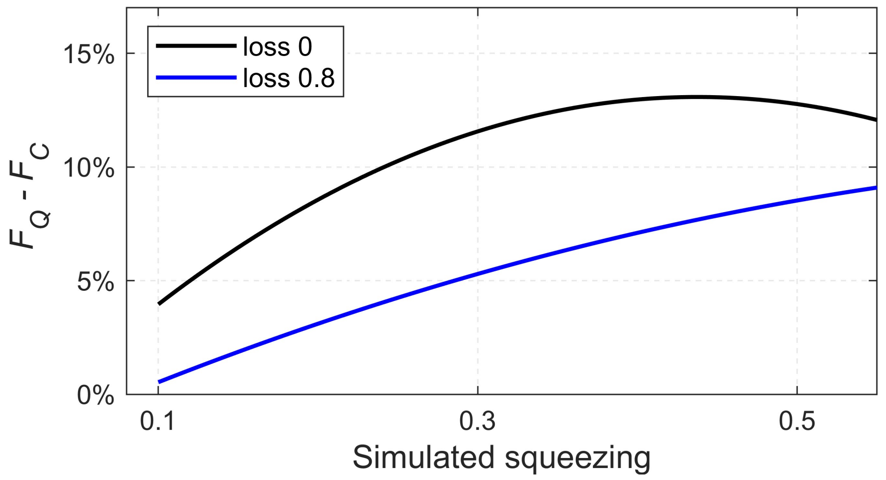

Supplementary Figure 8: Simulated fidelity difference between quantum and classical methods versus squeezing values under different loss values for formic acid molecules.

Supplementary Note 8: Derivation of Non-Condon effects

$$\hat{\mu} = \mu^{(0)} + \sum_{i=1}^{m} \mu_i^{(1)} \hat{Q}_i + ... \tag{43}$$

where $\mu_i^{(1)}$ is the first derivative with respect to the ith normal coordinate(Q_i) at the equilibrium structure of the initial state.

In Ref. [20], Huh. $et\,al.$ propose a theoretical model for simulating the non-Condon spectrum for the linear and quadratic HT terms with a boson sampling approach. They first introduce an auxiliary function,

$$f_{m'}(\kappa) = |\langle \mathbf{m}^{'} | \widehat{U}_{Dok} \exp(\kappa\hat{\mu}) | \mathbf{0} \rangle|^2, \tag{44}$$

where $\hat{\mu} = \hat{\mu}^{\dagger}$ and $\kappa \in \mathbb{C}$ to approximate the non-Condon profile. By Taylor-expanding the auxiliary function, a particular linear combination of different auxiliary functions results in an approximation of the non-Condon transition with a quadratically scaling error, which is given by

$$\langle \mathbf{m}^{'} | \widehat{U}_{Dok} \hat{\mu} | \mathbf{0} \rangle|^2 = \frac{1}{2\tau^2} \left[\; f_{m'}(i\tau) + \tfrac{1}{2} f_{m'}(\tau) + \tfrac{1}{2} f_{m'}(-\tau) - 2 f_{m'}(0) \; \right] + \mathcal{O}\left(\tau^2\right), \tag{45}$$

where τ is a real positive number. In Eq. (45), $f_{\mathbf{m}'}(0) = |\langle \mathbf{m}^{'}|\hat{U}_{Dok}|\mathbf{0}\rangle|^2$, which is an FCP and three evaluations of $f_{m'}$, which are non-Condon profiles can be implemented in a boson sampling network. In the next step, we describe how $f_{m'}$ can be expressed with Gaussian operators and further be implemented in the linear optical networks.

For the first-order HT expansion,

$$\begin{aligned} f_{m'}(\kappa) &= \exp\left(2\kappa\mu^{(0)}\right)\prod_{j=1}^{M}|C_{j,\kappa}|^2|\langle\mathbf{m}^{'}|\hat{U}_{Dok}\hat{D}(\boldsymbol{\alpha}_\kappa)|\mathbf{0}\rangle|^2 \\ &= \exp\left(2\kappa\mu^{(0)}\right)\prod_{j=1}^{M}|C_{j,\kappa}|^2|\langle\mathbf{m}^{'}|\hat{D}_{\mathbf{K}/\sqrt{2}}\hat{S}^{\dagger}_{\Omega'}\hat{R}_U\hat{S}_{\Omega}\hat{D}(\boldsymbol{\alpha}_\kappa)|\mathbf{0}\rangle|^2 \\ &= \exp\left(2\kappa\mu^{(0)}\right)\prod_{j=1}^{M}|C_{j,\kappa}|^2|\langle\mathbf{m}^{'}|\hat{R}_{C_L}\hat{S}^{\dagger}_{\Sigma}\hat{R}^{\dagger}_{C_R}\hat{D}_{J^{-1}\mathbf{K}/\sqrt{2}}\hat{D}(\boldsymbol{\alpha}_\kappa)|\mathbf{0}\rangle|^2 \\ &= \exp\left(2\kappa\mu^{(0)}\right)\prod_{j=1}^{M}|C_{j,\kappa}|^2|\langle\mathbf{m}^{'}|\hat{R}_{C_L}\hat{S}^{\dagger}_{\Sigma}\hat{R}^{\dagger}_{C_R}\hat{D}_{\boldsymbol{\alpha}_\kappa+J^{-1}\mathbf{K}/\sqrt{2}}|\mathbf{0}\rangle|^2 \\ &= \exp\left(2\kappa\mu^{(0)}\right)\prod_{j=1}^{M}|C_{j,\kappa}|^2|\langle\mathbf{m}^{'}|\hat{D}_{J\left(\boldsymbol{\alpha}_\kappa+J^{-1}\mathbf{K}/\sqrt{2}\right)}\hat{R}_{C_L}\hat{S}^{\dagger}_{\Sigma}\hat{R}^{\dagger}_{C_R}|\mathbf{0}\rangle|^2, \end{aligned} \tag{46}$$

where J, α_κ, $\mathbf{K}$, $\Sigma^{'}$, Σ, U are extracted from the molecular structure itself. The form shown in the last line of **Eq. (46)** is consistent with the original FCF form in Ref. [6], which is indeed a loop hafnian. From our previous derivation, the loop hafnian can be written as the sum of hafnians, and further be achieved in an experiment by the linear network.

For the second-order HT expansion, initially, we have,

$$f_{m'}(\kappa) = \exp\left(2\kappa\mu^{(0)}\right)\prod_{j=1}^{M}|C_{j,\kappa}|^2|\langle\mathbf{m}^{'}|\hat{R}(U_2)\hat{S}(\ln(L))\hat{R}(U_1)\hat{D}(\boldsymbol{\beta})\hat{R}\left(U^t\right)\hat{S}(\Xi_\kappa)\hat{D}(\alpha_\kappa)|\mathbf{0}\rangle|^2. \tag{47}$$

The Gaussian operator $\prod_{j=1}^{M}\hat{R}(U_2)\hat{S}(\ln(L))\hat{R}(U_1)\hat{D}(\boldsymbol{\beta})$ is the Condon approximation and $\prod_{j=1}^{M}\hat{R}(U^t)\hat{S}(\Xi_\kappa)\hat{D}(\alpha_\kappa)$ is the transition moment operator. Let $\hat{O}(\kappa) = \prod_{j=1}^{M}\hat{R}(U_2)\hat{S}(\ln(L))\hat{R}(U_1)\hat{D}(\boldsymbol{\beta})\hat{R}(U^t)\hat{S}(\Xi_\kappa)\hat{D}(\alpha_\kappa)$. To rewrite the Gaussian operator $\hat{O}(\kappa)$ in a simpler form, the Bloch-Messiah decomposition is used. The first step is to find X_κ, Y_κ and $\boldsymbol{z}_\kappa$ such that,

$$\hat{a}^{'\dagger} = \hat{O}^{\dagger}_{\kappa}\hat{a}^{\dagger}\hat{O}_\kappa = X_\kappa\hat{a} + Y_\kappa\hat{a}^{\dagger} + \boldsymbol{z}_\kappa, \tag{48}$$

where,

$$X_\kappa = U_2\sinh(\ln(L))U_1\boldsymbol{U}^t\cosh(\Xi_\kappa) + U_2\cosh(\ln(L))U_1U^t\sinh(\Xi_k), \tag{49}$$

$$Y_\kappa = U_2\cosh(\ln(L))U_1\boldsymbol{U}^t\cosh(\Xi_\kappa) + U_2\sinh(\ln(L))U_1U^t\sinh(\Xi_k), \tag{50}$$

$$z_\kappa = U_2 L U_1 U^t \exp\left(\Xi_k\right)\alpha_k + U_2 L U_1 U \beta. \tag{51}$$

Then, by taking the singular value decomposition $X_\kappa = V_\kappa \sinh\left(\Sigma_\kappa\right) W_\kappa^t$, $Y_\kappa = V_\kappa \cosh\left(\Sigma_\kappa\right) W_\kappa^\dagger$, we will have

$$f_{m'}\left(\kappa\right) = \exp\left(2\kappa\mu^{(0)}\right) \prod_{j=1}^{M} |C_{j,\kappa}|^2 |\langle \mathbf{m}' | \hat{D}\left(z_\kappa\right) \hat{R}\left(V_\kappa\right) \hat{S}\left(\Sigma_\kappa\right) \hat{R}\left(W_\kappa\right)^\dagger |\mathbf{0}\rangle|^2. \tag{52}$$

Similarly, this operator form can also be converted into the expression of summation of hafnian, which is realized experimentally.

Supplementary Note 9: Vibrational excitation simulation

During molecular simulation, FCFs are used not only in studying the molecular vibronic spectra but also for other simulations, such as molecular vibrational excitations during vibronic transitions [21] and single-molecule electron transport [22]. **Supplementary Figure 9** depicts the experimental demonstration of vibrational excitations in pyrrole during photoexcitation-mediated vibronic processes. For instance, we examine the vibrational excitation of six out of 24 normal modes in pyrrole's excited electronic state, both with and without pre-excitation of the oxygen-hydrogen (O-H) stretching mode at the ground electronic state (detailed in **Supplementary Note 5**). From the marginal distributions, the initial pre-excitation is transferred to two vibrational modes of the excited electronic state with the vibrational frequencies of 3231.5 cm^{-1} and 3250.8 cm^{-1} due to the Duschinsky mode mixing. This proof-of-principle demonstration shows the possibility of predicting the vibrational excitation of molecules in vibronic processes using our quantum microprocessor, which may help the predictions of molecular dynamics simulations in the future.

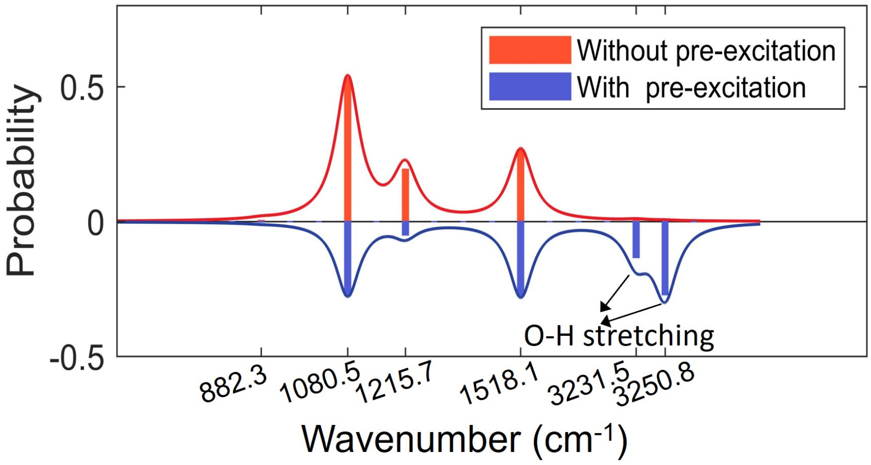

Supplementary Figure 9: Vibrational excitation simulations. Single-mode marginal distribution of pyrrole during a vibronic transition from ground to the first excited state without (top) and with (bottom) pre-excitation of the ground state O–H stretching mode of pyrrole.

References

[1] Michael A Nielsen and Isaac Chuang. Quantum computation and quantum information, 2002.

[2] Marlan O Scully and M Suhail Zubairy. Quantum optics, 1999.

[3] Alex DD Craik. Prehistory of faà di bruno's formula. *The American Mathematical Monthly*, 112(2):119–130, 2005.

[4] Regina Kruse, Craig S Hamilton, Linda Sansoni, Sonja Barkhofen, Christine Silberhorn, and Igor Jex. Detailed study of gaussian boson sampling. *Physical Review A*, 100(3):032326, 2019.

[5] H-C Jankowiak, JL Stuber, and R Berger. Vibronic transitions in large molecular systems: Rigorous prescreening conditions for franck-condon factors. *The Journal of chemical physics*, 127(23), 2007.

[6] Nicolás Quesada. Franck-condon factors by counting perfect matchings of graphs with loops. *The Journal of chemical physics*, 150(16):164113, 2019.

[7] Robert Berger and Martin Klessinger. Algorithms for exact counting of energy levels of spectroscopic transitions at different temperatures. *Journal of computational chemistry*, 18(10):1312–1319, 1997.

[8] Sohang Kundu, Partha Pratim Roy, Graham R Fleming, and Nancy Makri. Franck–condon and herzberg–teller signatures in molecular absorption and emission spectra. *The Journal of Physical Chemistry B*, 126(15):2899–2911, 2022.

[9] F. Duschinsky. The importance of the electron spectrum in multi atomic molecules. concerning the franck-condon principle. *Acta Physicochim. URSS*, 7:551–566, 1937.

[10] Joonsuk Huh, Gian Giacomo Guerreschi, Borja Peropadre, Jarrod R McClean, and Alán Aspuru-Guzik. Boson sampling for molecular vibronic spectra. *Nature Photonics*, 9(9):615–620, 2015.

[11] E. V. Doktorov, I. A. Malkin, and V. I. Man'ko. Dynamical symmetry of vibronic transitions in polyatomic molecules and the franck-condon principle. *Journal of Molecular Spectroscopy*, 64(2):302–326, 1977.

[12] Jacob FF Bulmer, Bryn A Bell, Rachel S Chadwick, Alex E Jones, Diana Moise, Alessandro Rigazzi, Jan Thorbecke, Utz-Uwe Haus, Thomas Van Vaerenbergh, Raj B Patel, et al. The boundary for quantum advantage in gaussian boson sampling. *Science advances*, 8(4):eabl9236, 2022.

[13] Leonid Gurvits. On the complexity of mixed discriminants and related problems. In *Mathematical Foundations of Computer Science 2005: 30th International Symposium, MFCS 2005, Gdansk, Poland, August 29–September 2, 2005. Proceedings 30*, pages 447–458. Springer, 2005.

[14] Scott Aaronson and Alex Arkhipov. The computational complexity of linear optics. In *Proceedings of the forty-third annual ACM symposium on Theory of computing*, pages 333–342, 2011.

[15] Scott Aaronson. Chinese bosonsampling experiment: the gloves are off, 2020.

[16] Changhun Oh, Youngrong Lim, Yat Wong, Bill Fefferman, and Liang Jiang. Quantum-inspired classical algorithms for molecular vibronic spectra. *Nature Physics*, pages 1–7, 2024.

[17] Michael Reck, Anton Zeilinger, Herbert J Bernstein, and Philip Bertani. Experimental realization of any discrete unitary operator. *Physical review letters*, 73(1):58, 1994.

[18] Stefano Paesani, Massimo Borghi, Stefano Signorini, Alexandre Maïnos, Lorenzo Pavesi, and Anthony Laing. Near-ideal spontaneous photon sources in silicon quantum photonics. *Nature communications*, 11(1):2505, 2020.

[19] Andreas Christ, Kaisa Laiho, Andreas Eckstein, Katiúscia N Cassemiro, and Christine Silberhorn. Probing multimode squeezing with correlation functions. *New Journal of Physics*, 13(3):033027, 2011.

[20] Hamza Jnane, Nicolas PD Sawaya, Borja Peropadre, Alan Aspuru-Guzik, Raul Garcia-Patron, and Joonsuk Huh. Analog quantum simulation of non-condon effects in molecular spectroscopy. *ACS Photonics*, 8(7):2007–2016, 2021.

[21] Soran Jahangiri, Juan Miguel Arrazola, Nicolás Quesada, and Alain Delgado. Quantum algorithm for simulating molecular vibrational excitations. *Physical Chemistry Chemical Physics*, 22(44):25528–25537, 2020.

[22] Soran Jahangiri, Juan Miguel Arrazola, and Alain Delgado. Quantum algorithm for simulating single-molecule electron transport. *The Journal of Physical Chemistry Letters*, 12(4):1256–1261, 2021.
